# Fault Detection Filter Design and Optimization for Switched Systems with All Modes Unstable

**DOI:** 10.1155/2022/8339634

**Published:** 2022-04-04

**Authors:** Hanqiao Huang, Haoyu Cheng, Ruijia Song, Gonghao Sun, Yangwang Fang, Guan Huang

**Affiliations:** ^1^Unmanned System Research Institute, Northwestern Polytechnical University, Xi'an, China; ^2^School of Astronautics, Northwestern Polytechnical University, Xi'an, China; ^3^Shanghai Electro-Mechanical Engineering Institute, Shanghai, China; ^4^China Electronics Standardization Institute, Beijing, China

## Abstract

This problem of intelligent switched fault detection filter design is investigated in this article. Firstly, the mode-dependent average dwell time (MDADT) method is applied to generate the time-dependent switching signal for switched systems with all subsystems unstable. Afterwards, the switched fault detection filter is proposed for the generation of residual signal, which consists of dynamics-based filter and learning-based filter. The MDADT method and multiple Lyapunov function (MLF) method are employed to guarantee the stability and prescribed attenuation performance. The parameters of dynamics-based filter are given by solving a series of linear matrix inequalities. To improve the transient performance, the deep reinforcement learning is introduced to design learning-based filter in the framework of actor-critic. The output of learning-based filter can be viewed as uncertainties of dynamics-based filter. The deep deterministic policy gradient algorithm and nonfragile control are adopted to guarantee the stability of algorithm and compensate the external disturbance. Finally, simulation results are given to illustrate the effectiveness of the method in the paper.

## 1. Introduction

Switched systems [1–3] are an important component of hybrid systems, which are composed of a series of subsystems and build the connection between nonlinear systems and linear systems. As an efficient way to deal with complex nonlinear systems, switched systems have attacked considerable attentions and were applied in the areas of both military and economics [4, 5], such as industrial manufacturing, flight control, robotic control, process control, and so on. The problems of modeling, stability analysis [6], controller design [7], and filter design [8] have been investigated recently and fruitful encouraging results emerged [9]. To mention a few, the problem of switched fault tolerant controller design is investigated in [10], the event-triggered controller design method for discrete-time switched systems is proposed in [11], and the stability and *l*_2_-gain analysis are given in [12].

The basic problem for switched systems lies in stability analysis [13, 14]. The scholars have developed tools to analyze the stability in past decades, such as common Lyapunov function (CLF) method, MLF method, and persistence Lyapunov function method [15]. The CLF method is mainly applied for the switched systems with arbitrary switching, which means that the switched systems are stable if all the subsystems share a CLF. However, it is difficult to design a CLF for all subsystems. To obtain tighter bounds, the stability analysis can be developed by the aid of MLF, which is mainly applied to constrained switching. Moreover, in practical system, the switching signal always depends on state or time. Because it is difficult to obtain the state measurements, the switching signal is time-dependent in many situations. Thus, the average dwell time (ADT) method and MDADT method provide efficient solutions to deal with the stability analysis for switched systems with constrained switching. In the work of [16], the issue of stability analysis for switched positive linear systems is studied. The ADT method and multiple linear copositive Lyapunov function are combined and sufficient stability criteria for stability analysis are given. However, it is straightforward that common parameters for all subsystems with different characteristic will lead to conservativeness, which motivates the researches on MDADT. The definition of MDADT is firstly proposed in [17]. The problem of stability analysis is studied in the framework of MDADT method and tighter bounds are obtained. In [18], the problem of stability and robust controller design for switched systems with external disturbances is studied. The MDADT switching and MLF method are introduced to ensure the stability. Moreover, in practice, the error of the switched system will lead to unstability. Therefore, the researches of stability analysis for switched systems with unstable subsystems are still one of the most important topics in control areas. In [19], the piecewise Lyapunov functions and MDADT method are combined to deal with the problem of stability analysis for switched systems with unstable modes. The fast switching is applied to unstable modes and slow switching is applied to stable modes. In [20], the problem of stability for switched systems with input time delay is studied. The unstable subsystems and asynchronous switching caused by time delay are taken into consideration. The extended stability criteria are obtained by the aid of Lyapunov-Krasovskii function method and sufficient conditions for stability analysis are presented.

The fault case of system will no doubt lead to undesirable response [21, 22]. One issue in the industrial systems or aeronautic engineering is that the maintenance cannot be given immediately to ensure the reliability and safety [23]. Thus, the presence of undesirable fault and the possibility of the occurrence of faults has to be considered in the stability analysis and system design, which motivates the study on fault detection and fault tolerant control. As an efficient way to deal with the undesirable faults, fault detection technique has attracted more and more attention, which can be seen in the recent important results, such as [24–26]. The fault detection system can detect the fault in time and efficiently so that we can reconstruct the control diagram to adjust to the fault environment. Among these fault detection methods, the most valuable and applicable method is the model-based fault detection filter design method. It can be inferred in existing literature that the model-based method consists of a residual generator and residual evaluator, which is applied in the areas of networked control systems, aerospace engineering, and process control. In [27], the problem of *H*_∞_/*H*-fault detection for switched systems with unstable modes is investigated. The robustness to external disturbance and the sensitivity to fault are both taken into consideration. In [28], the problem of cooperative control for multiagent systems is investigated based on adaptive control and fuzzy control. The unknown control parameters and actuator fault are taken into consideration. The backstepping control is utilized to derive the controller. In the work of [29], the problem of fault detection subject to nonlinearities and disturbance is investigated according to event-triggered scheme. Sufficient conditions to guarantee the system is stable with the prescribed performance are obtained based on ADT method and MLF method. Based on the literature mentioned above, it can be inferred that the model-based fault detection filter is popularly applied in most of engineering problems because of the design simplicity and physical execution. However, it is difficult to achieve optimal compromise between robustness and transient performance. The model-based method can guarantee the stability and robustness of the closed-loop system. But the transient performance cannot be guaranteed. It is essential and significant to improve the transient performance of fault detection filters. Moreover, due to the lack of online-learning ability, this type of fault detection filters is relatively ineffective to have optimal performance in the real-world uncertain environment.

With the development of machine learning and computer science, the intelligent control has drawn considerable attention [30]. As a powerful nonlinear approximation approach, deep learning and deep reinforcement learning has promoted the considerable performance in realistic applications, such as controller parameters tuning, decision making, and so on. In particular, the DDPG algorithm has been illustrated to perform stably and efficiently on many high-dimensional action control tasks. In the work of [31], a noninteger PID controller based on DDPG algorithm is proposed for the tracking problem. To accomplish the control methodology, a kinematic controller and a dynamic controller are established independently, in which the kinematic controller is proposed based on the model of the vehicle and the dynamic controller is realized for the supplementary kinematic controller to achieve optimal performance. In [32], the assembly task is defined as a Markov decision process and a fuzzy DDPG algorithm is given to realize the task. To improve the performance and learning efficiency, a fuzzy reward system is developed for the assembly task. In addition, in the work of [33–35], the machine learning is applied in the design of controller and guidance law.

Inspired by the aforementioned discussion, an effort is conducted in the paper to design an intelligent fault detection filter for switched system with all modes unstable, which is composed of dynamics-based filter and learning-based filter. As well known, the model-based fault detection filter is designed in the existing literature, which can guarantee the stability of closed-loop system and the robustness to external disturbance. However, how to improve the transient performance of fault detection still remains an open problem. On the other hand, in most literature, the fault detection filter is proposed based on the assumption that all the subsystems are stable. But in many practical situations, the subsystems may be unstable, which motivates the study in this paper. The robust control theory is applied to establish the dynamics-based filter. The switched system with all modes unstable is considered and the fault detection filter is presented for generation of the residual signal. The MDADT method and MLF method are combined to ensure the stability and prescribed attenuation performance index of switched system. To achieve optimal performance, the learning-based filter is introduced based on DDPG algorithm in the framework of actor-critic, in which the filter parameters are optimized by online learning. The output of learning-based filter can be viewed as variation of the parameters of dynamics-based filter. Thus, the nonfragile control theory is introduced to guarantee the stability of switched systems. The main contributions of this article are stated as follows: (1) An observer-based filter is proposed to deal with the problem of fault detection for switched system with all modes unstable, in which the stability and attenuation performance index can be guaranteed by MDADT method and MLF method. Compared to the existing results, the characteristic of each subsystem is considered, and tighter bounds can be obtained, which provides more room for the improvement of flexibility. (2) The learning-based fault detection filter is presented based on DDPG algorithm to achieve optimal performance, which overcomes the undesirable response caused by external disturbance and uncertainties. The action is defined by the variation of parameters of the fault detection filter. (3) The nonfragile control theory is applied to ensure the stability of closed-loop system, for the reason that the output of actor network is viewed as the variation of parameters of dynamics-based filter.

The rest of the paper is presented as follows: the model of switched system with all modes unstable is established in Section 2, in which the fault detection filter is proposed to generate the residual signal. In Section 2, the main results of fault detection are proposed, which is composed of dynamics-based filter and learning-based filter. The numerical example is given in Section 4 to validate the effectiveness of proposed method, which is followed by the conclusion in Section 5.

## 2. Problem Statement

The continuous-time switched system in this paper can be described as follows:

(1) $$\begin{array}{c} \left\{\begin{array}{c} \dot{x}\left(t\right)=A_{\sigma \left(t\right)}x\left(t\right)+B_{\sigma \left(t\right)}u\left(t\right)+D_{\sigma \left(t\right)}\omega \left(t\right)+F_{\sigma \left(t\right)}f\left(t\right),\\ y\left(t\right)=C_{\sigma \left(t\right)}x\left(t\right). \end{array}\right. \end{array}$$

*x*(*t*) is the state vector; *u*(*t*) denotes the input signal; *y*(*t*) is the output signal; *ω*(*t*) ∈ *L*_2_[0, *∞*) is the external disturbances; *f*(*t*) represents the fault signal to be detected; *i*=*σ*(*k*) : [0, *∞*)⟶*N*={1,2, ..., *n*} is piecewise switching signal; *A*_*σ*(*t*)_, *B*_*σ*(*t*)_, *C*_*σ*(*t*)_, and *F*_*σ*(*t*)_ are system matrices with appropriate dimensions.

To improve the transient performance of fault detection filter and achieve optimal performance, it is supposed that the fault detection filter in this paper is composed of two parts: the dynamics-based filter and the learning-based filter. The dynamics-based filter is designed according to robust control theory and the learning-based filter is proposed based on deep reinforcement learning, which can be described as follows:

(2) $$\begin{array}{c} L_{\sigma \left(t\right)}=L_{\text{r,}\sigma \left(t\right)}+L_{\text{d},\sigma \left(t\right)}. \end{array}$$

*L*_r,*σ*(*t*)_ is the parameters of dynamics-based fault detection filter, which is determined by robust control theory; *L*_d,*σ*(*t*)_ is the compensation for external disturbance, which is generated by the aid of DDPG algorithm. To ensure the stability of DDPG algorithm, the compensation can be viewed as the variation of parameters. Thus, the stability of optimization algorithm is guaranteed by nonfragile control theory. The parameters obtained by DDPG algorithm can be written as

(3) $$\begin{array}{c} L_{\text{d},\sigma \left(t\right)}=\Delta L_{i}=M_{i}E_{i}N_{i}. \end{array}$$

*M*_*i*_ and *N*_*i*_ are the known matrices with appropriate dimensions; *E*_*i*_ are unknown matrices satisfying *E*_*i*_^T^*E*_*i*_ ≤ *I*.

**Remark 1 .**

The designing process can be divided into two steps: (1) regarding the design of robust control theory, it is proposed to ensure the stability of closed-loop system and prescribed attenuation performance. (2) The deep reinforcement learning is utilized to improve the performance of fault detection, where the additional compensation of fault detection filter is viewed as the action of the agent. Then the parameters of fault detection filter are optimized based on DDPG algorithm in the framework of actor-critic. Compared with the traditional methods, not only can the stability be guaranteed by robust control theory, but also the transient performance of fault detection can be improved based on DDPG algorithm.

**Remark 2 .**

The problem of fault detection for switched systems with all modes unstable is studied in this paper. The unstable modes are taken into consideration and it is more applicable. The MDADT method and MLF method are combined to analyze the stability and tighter bounds on dwell time can be obtained than the traditional ADT method. There is more room for the switched systems to dwell long enough time to decrease the system energy. Therefore, the method proposed in this paper will improve the design flexibility of fault detection system.

The dynamics-based fault detection filter is proposed to generate the residual signal, which can be described as follows:

(4) $$\begin{array}{c} \left\{\begin{array}{c} \dot{\hat{x}}\left(t\right)=A_{\sigma \left(t\right)}\hat{x}\left(t\right)+B_{\sigma \left(t\right)}u\left(t\right)+L_{\text{r},\sigma \left(t\right)}\left(y\left(t\right)-\hat{y}\left(t\right)\right),\\ \hat{y}\left(t\right)=C_{\sigma \left(t\right)}\hat{x}\left(t\right),\\ r\left(t\right)=y\left(t\right)-\hat{y}\left(t\right), \end{array}\right. \end{array}$$

where $\hat{x}\left(t\right)$ is the state of fault detection filter; $\hat{y}\left(t\right)$ denotes the estimation of output signal *y*(*t*); *r*(*t*) is the residual signal and *L*_*σ*(*t*)_ denote the parameter matrices of fault detection filter to be determined.

Define the error of state measurement as $e\left(t\right)=x\left(t\right)-\hat{x}\left(t\right)$; the error of fault estimation $\tilde{r}\left(t\right)=r\left(t\right)-f\left(t\right)$. Thus, we set the augmented state vector $\tilde{x}\left(t\right)={\left[\begin{array}{cc} x^{\text{T}}\left(t\right) & e^{\text{T}}\left(t\right) \end{array}\right]}^{\text{T}}$ and the augmented input signal as $\tilde{\omega }\left(t\right)={\left[\begin{array}{ccc} u^{\text{T}}\left(t\right) & \omega^{\text{T}}\left(t\right) & f^{\text{T}}\left(t\right) \end{array}\right]}^{\text{T}}$, so we can obtain the closed-loop switched system as follows:

(5) $$\begin{array}{c} \left\{\begin{array}{c} \dot{\tilde{x}}\left(t\right)={\tilde{A}}_{i}\tilde{x}\left(t\right)+{\tilde{B}}_{i}\tilde{\omega }\left(t\right),\\ \tilde{r}\left(t\right)={\tilde{C}}_{i}\tilde{x}\left(t\right)+{\tilde{D}}_{i}\tilde{\omega }\left(t\right), \end{array}\right. \end{array}$$

where ${\tilde{A}}_{i}=\left[\begin{array}{cc} A_{i} & 0\\ 0 & A_{i}-L_{i}C_{i} \end{array}\right]$, ${\tilde{B}}_{i}=\left[\begin{array}{ccc} B_{i} & D_{i} & F_{i}\\ 0 & D_{i} & F_{i} \end{array}\right]$, ${\tilde{C}}_{i}=\left[\begin{array}{cc} 0 & C_{i} \end{array}\right]$, ${\tilde{D}}_{i}=\left[\begin{array}{ccc} 0 & 0 & -I \end{array}\right]$.

Then we can conclude that the design of robust fault detection filter can be converted by the problem of *H*_∞_ filter design. Therefore, for given prescribed attenuation performance index *γ*_*w*_, the closed-loop switched system in equation (5) is globally uniformly exponentially stable (GUES) when $\tilde{\omega }\left(t\right)=0$; the following inequality holds for all nonzero $\tilde{\omega }\left(t\right)$ under zero-initial condition.

(6) $$\begin{array}{c} \int_{0}^{\infty }{\tilde{r}}^{\text{T}}\left(s\right)\tilde{r}\left(s\right)\text{d}s\leq \gamma^{2}\int_{0}^{\infty }{\tilde{\omega }}^{\text{T}}\left(s\right)\tilde{\omega }\left(s\right)\text{d}s. \end{array}$$

The residual signal is generated by fault detection filter; it is necessary to design a residual evaluator, which consists of an evaluation function and a threshold. The evaluation function and threshold can be written as

(7) $$\begin{array}{c} J\left(t\right)=\left(\frac{1}{\eta }\int_{s=t-\eta }^{t}r^{T}\left(s\right)r\left(s\right)\right),\\ J_{th}=\underset{\omega \in L_{2}\left[0,\infty \right),\text{f}=0}{\sup}J\left(k\right). \end{array}$$

*η* denotes the length of time window of evaluation function.

Based on residual evaluator and threshold, the decision logic can be expressed as follows:

(8) $$\begin{array}{c} \left\{\begin{array}{c} \left\|J_{th}\right\|\geq \left\|J\left(t\right)\right\|,\text{no fault}\Rightarrow \text{no alarm,}\\ \left\|J_{th}\right\|<\left\|J\left(t\right)\right\|,\text{fault}\Rightarrow \text{alarm.} \end{array}\right. \end{array}$$

## 3. Main Results

### 3.1. Robust Filter Design

The definitions and lemmas are given as follows to make the proof convenient.

**Definition 1 .**

[17]. For given switching logic *σ*(*t*) and time interval [*t*_0_, *t*_1_], we define *N*_*σ*,*i*_(*t*_0_, *t*_1_) as the number of switching during the time interval [*t*_0_, *t*_1_]. If there exist constants *N*_0*i*_ ≥ 0 and *τ*_*ai*_ > 0, such that

(9) $$\begin{array}{c} N_{\sigma ,i}\left(t_{0},t_{1}\right)\geq N_{0i}+\frac{T_{i}\left(t_{0},t_{1}\right)}{\tau_{ai}}. \end{array}$$

Then, *τ*_*ai*_ is called the MDADT of fast switching and *N*_0*i*_ is called the chattering bounds; we set *N*_0*i*_=0 in this paper.

**Definition 2 .**

(see [12]). For given switching logic *σ*(*t*), if there exist constant scalars *δ* > 0, *ε* > 0, and equation (10) holds for $\tilde{\omega }\left(t\right)=0$, then the switched system in (5) is GUES.

(10) $$\begin{array}{c} \left\|e\left(t\right)\right\|\leq \delta e^{-\varepsilon \left(t-t_{0}\right)}\left\|e\left(t_{0}\right)\right\|. \end{array}$$

**Lemma 1 .**

(see [27]). For given matrices *S*, *T* and symmetric matrix *Y*, if there exist constant scalar *κ* > 0, such that

(11) $$\begin{array}{c} Y+\kappa^{-1}S^{\text{T}}S+\kappa T^{\text{T}}T<0. \end{array}$$

then for matrix *E*_*i*_ with *E*_*i*_^T^*E*_*i*_ ≤ *I*, we have

(12) $$\begin{array}{c} Y+S^{\text{T}}ET+T^{\text{T}}E^{\text{T}}S<0, \end{array}$$

**Theorem 1 .**

For given constant scalars 0 < *μ*_*i*_ < 1, *λ*_*i*_ > 0, if there exist positive definite matrices *P*_*i*_, such that

(13) $$\begin{array}{c} P_{i}\leq \mu_{i}P_{j},\forall i\neq j, \end{array}$$

(14) $$\begin{array}{c} {\tilde{A}}_{i}^{\text{T}}\leq P_{i}+P_{i}{\tilde{A}}_{i}\leq \lambda_{i}P_{i} \end{array}$$

then the switched system in equation (5) is GUES if MDADT satisfies the following equation:

(15) $$\begin{array}{c} 0\leq \tau_{ai}\leq \tau_{ai}^{*}=-\frac{\ln\mu_{\sigma \left(t_{i}\right)}}{\lambda_{\sigma \left(t_{i}\right)}}. \end{array}$$

**Proof**

We set the Lyapunov function as follows:

(16) $$\begin{array}{c} V_{i}\left(\tilde{x}\left(t\right)\right)={\tilde{x}}^{\text{T}}\left(t\right)P_{i}\tilde{x}\left(t\right). \end{array}$$

Thus, we have

(17) $$\begin{array}{c} {\dot{V}}_{i}\left(t\right)-\lambda_{i}V_{i}\left(t\right)\\ ={\left[\begin{array}{c} \tilde{x}\left(t\right)\\ \tilde{\omega }\left(t\right) \end{array}\right]}^{\text{T}}{\left[\begin{array}{cc} {\tilde{A}}_{i} & {\tilde{B}}_{i} \end{array}\right]}^{\text{T}}P_{i}\tilde{x}\left(t\right)+{\tilde{x}}^{\text{T}}\left(t\right)P_{i}\left[\begin{array}{cc} {\tilde{A}}_{i} & {\tilde{B}}_{i} \end{array}\right]\left[\begin{array}{c} \tilde{x}\left(t\right)\\ \tilde{\omega }\left(t\right) \end{array}\right]-\lambda_{i}{\tilde{x}}^{\text{T}}\left(t\right)P_{i}\tilde{x}\left(t\right)\\ ={\left[\begin{array}{c} \tilde{x}\left(t\right)\\ \tilde{\omega }\left(t\right) \end{array}\right]}^{\text{T}}\left[\begin{array}{cc} {\tilde{A}}_{i}^{\text{T}}P_{i}+P_{i}{\tilde{A}}_{i}-\lambda_{i}P_{i} & P_{i}{\tilde{B}}_{i}\\ {}* & 0 \end{array}\right]\left[\begin{array}{c} \tilde{x}\left(t\right)\\ \tilde{\omega }\left(t\right) \end{array}\right]. \end{array}$$

Combining equation (14) and $\tilde{\omega }\left(t\right)=0$, we can conclude that

(18) $$\begin{array}{c} {\dot{V}}_{i}\left(t\right)-\lambda_{i}V_{i}\left(t\right)={\tilde{x}}^{\text{T}}\left(t\right)\left({\tilde{A}}_{i}^{\text{T}}P_{i}+P_{i}{\tilde{A}}_{i}-\lambda_{i}P_{i}\right)\tilde{x}\left(t\right)\leq 0. \end{array}$$

It is supposed that the switching instants during time interval [0, *t*] are set to be *t*_1_, *t*_2_,…，*t*_*k*_ with *t*_*k*+1_ = *t*; we can derive that

(19) $$\begin{array}{c} V_{\sigma \left(t\right)}\left(t\right)\leq e^{\lambda_{\sigma \left(t\right)}\left(t-t_{k}\right)}V_{\sigma \left(t\right)}\left(t_{k}\right)\\ \leq \mu_{\sigma \left(t\right)}e^{\lambda_{\sigma \left(t\right)}\left(t-t_{k}\right)}V_{\sigma \left(t_{k}\right)}\left(t_{k}^{-}\right)\\ \leq \mu_{\sigma \left(t\right)}e^{\lambda_{\sigma \left(t\right)}\left(t-t_{k}\right)}e^{\lambda_{\sigma \left(t_{k}\right)}\left(t_{k}-t_{k-1}\right)}V_{\sigma \left(t_{k}\right)}\left(t_{k-1}\right)\\ \leq \mu_{\sigma \left(t\right)}\mu_{\sigma \left(t_{k}\right)}e^{\lambda_{\sigma \left(t\right)}\left(t-t_{k}\right)}e^{\lambda_{\sigma \left(t_{k}\right)}\left(t_{k}-t_{k-1}\right)}V_{\sigma \left(t_{k-1}\right)}\left(t_{k-1}^{-}\right)\\ \cdot \cdot \cdot \\ \leq \overset{k}{\underset{s=1}{\prod }}\mu_{\sigma \left(t_{s}\right)}e^{\overset{k}{\underset{s=1}{\sum }}\lambda_{\sigma \left(t_{s}\right)}\left(t_{s+1}-t_{s}\right)}V_{\sigma \left(0\right)}\left(0\right). \end{array}$$

Together with Definition 1, we have

(20) $$\begin{array}{c} V_{\sigma \left(t\right)}\left(t\right)\leq \exp\left[\sum_{i=1}^{k}N_{\sigma ,i}\left(0,t\right)\ln\mu_{\sigma \left(t_{i}\right)}+\sum_{i=1}^{k}T_{i}\left(0,t\right)\lambda_{\sigma \left(t_{i}\right)}\right]V_{\sigma \left(0\right)}\left(0\right)\\ \leq \exp\left[\sum_{i=1}^{k}\frac{T_{i}\left(0,t\right)}{\tau_{ai}}\ln\mu_{\sigma \left(t_{i}\right)}+\sum_{i=1}^{k}T_{i}\left(0,t\right)\lambda_{\sigma \left(t_{i}\right)}\right]V_{\sigma \left(0\right)}\left(0\right)\\ \leq \exp\left[\sum_{i=1}^{k}\left(\frac{\ln\mu_{\sigma \left(t_{i}\right)}}{\tau_{ai}}+\lambda_{\sigma \left(t_{i}\right)}\right)T_{i}\left(0,t\right)\right]V_{\sigma \left(0\right)}\left(0\right). \end{array}$$

If the MDADT of switched system satisfies equation (15), we have

(21) $$\begin{array}{c} \frac{\ln\mu_{\sigma \left(t_{i}\right)}}{\tau_{ai}}+\lambda_{\sigma \left(t_{i}\right)}\leq 0. \end{array}$$

Combining with Definition 2, one can conclude that the switched system in equation (5) is GUES when $\tilde{\omega }\left(t\right)=0$.

**Theorem 2 .**

For given constants 0 < *μ*_*i*_ < 1, *λ*_*i*_ > 0, *γ* > 0, if there exist positive definite matrices *P*_*i*_, such that

(22) $$\begin{array}{c} P_{i}\leq \mu_{i}P_{j},\forall i\neq j,\\ \left[\begin{array}{ccc} {\tilde{A}}_{i}^{\text{T}}P_{i}+P_{i}{\tilde{A}}_{i}-\lambda_{i}P_{i} & P_{i}{\tilde{B}}_{i} & {\tilde{C}}_{i}^{\text{T}}\\ {}* & -\gamma^{2}I & {\tilde{D}}_{i}^{\text{T}}\\ {}* & * & -I \end{array}\right]\leq 0. \end{array}$$

Then, the switched system with MDADT satisfying equation (15) is GUES with prescribed attenuation performance *γ*_*d*_, where *γ*_*d*_=*γ*^2^*μ*_min_^−*k*/2^.

**Proof**

Define the Lyapunov function in equation (16); we can obtain the following equations under zero-initial condition.

(23) $$\begin{array}{c} {\dot{V}}_{i}\left(t\right)-\lambda_{i}V_{i}\left(t\right)+{\tilde{r}}^{\text{T}}\left(t\right)\tilde{r}\left(t\right)-\gamma^{2}{\tilde{\omega }}^{\text{T}}\left(t\right)\tilde{\omega }\left(t\right)\\ ={\left[\begin{array}{c} \tilde{x}\left(t\right)\\ \tilde{\omega }\left(t\right) \end{array}\right]}^{\text{T}}\left[\begin{array}{cc} {\tilde{A}}_{i}^{\text{T}}P_{i}+P_{i}{\tilde{A}}_{i}-\lambda_{i}P_{i} & P_{i}{\tilde{B}}_{i}\\ {}* & 0 \end{array}\right]\left[\begin{array}{c} \tilde{x}\left(t\right)\\ \tilde{\omega }\left(t\right) \end{array}\right]\\ +{\left[\begin{array}{c} \tilde{x}\left(t\right)\\ \tilde{\omega }\left(t\right) \end{array}\right]}^{\text{T}}{\left[\begin{array}{cc} {\tilde{C}}_{i} & {\tilde{D}}_{i} \end{array}\right]}^{\text{T}}\left[\begin{array}{cc} {\tilde{C}}_{i} & {\tilde{D}}_{i} \end{array}\right]\left[\begin{array}{c} \tilde{x}\left(t\right)\\ \tilde{\omega }\left(t\right) \end{array}\right]-\gamma^{2}{\tilde{\omega }}^{\text{T}}\left(t\right)\tilde{\omega }\left(t\right)\\ ={\left[\begin{array}{c} \tilde{x}\left(t\right)\\ \tilde{\omega }\left(t\right) \end{array}\right]}^{\text{T}}\left(\left[\begin{array}{cc} {\tilde{A}}_{i}^{\text{T}}P_{i}+P_{i}{\tilde{A}}_{i}-\lambda_{i}P_{i} & P_{i}{\tilde{B}}_{i}\\ {}* & -\gamma^{2}I \end{array}\right]+{\left[\begin{array}{cc} {\tilde{C}}_{i} & {\tilde{D}}_{i} \end{array}\right]}^{\text{T}}\left[\begin{array}{cc} {\tilde{C}}_{i} & {\tilde{D}}_{i} \end{array}\right]\right)\left[\begin{array}{c} \tilde{x}\left(t\right)\\ \tilde{\omega }\left(t\right) \end{array}\right]. \end{array}$$

Based on Schur complement, we have

(24) $$\begin{array}{c} {\dot{V}}_{i}\left(t\right)-\lambda_{i}V_{i}\left(t\right)+{\tilde{r}}^{\text{T}}\left(t\right)\tilde{r}\left(t\right)-\gamma^{2}{\tilde{\omega }}^{\text{T}}\left(t\right)\tilde{\omega }\left(t\right)\\ ={\left[\begin{array}{c} \tilde{x}\left(t\right)\\ \tilde{\omega }\left(t\right) \end{array}\right]}^{\text{T}}\left[\begin{array}{ccc} {\tilde{A}}_{i}^{\text{T}}P_{i}+P_{i}{\tilde{A}}_{i}-\lambda_{i}P_{i} & P_{i}{\tilde{B}}_{i} & {\tilde{C}}_{i}^{\text{T}}\\ {}* & -\gamma^{2}I & {\tilde{D}}_{i}^{\text{T}}\\ {}* & * & -I \end{array}\right]\left[\begin{array}{c} \tilde{x}\left(t\right)\\ \tilde{\omega }\left(t\right) \end{array}\right]\\ \leq 0. \end{array}$$

According to the statement above, we have

(25) $$\begin{array}{c} V_{\sigma \left(t\right)}\left(t\right)\leq e^{\lambda_{\sigma \left(t\right)}\left(t-t_{k}\right)}V_{\sigma \left(t\right)}\left(t_{k}\right)-\int_{t_{k}}^{t}e^{\lambda_{\sigma \left(t\right)}\left(t-s\right)}\left({\tilde{r}}^{\text{T}}\left(s\right)\tilde{r}\left(s\right)-\gamma^{2}{\tilde{\omega }}^{\text{T}}\left(s\right)\tilde{\omega }\left(s\right)\right)\text{d}s\\ \leq \mu_{\sigma \left(t\right)}e^{\lambda_{\sigma \left(t\right)}\left(t-t_{k}\right)}V_{\sigma \left(t_{k}\right)}\left(t_{k}^{-}\right)-\int_{t_{k}}^{t}e^{\lambda_{\sigma \left(t\right)}\left(t-s\right)}\left({\tilde{r}}^{\text{T}}\left(s\right)\tilde{r}\left(s\right)-\gamma^{2}{\tilde{\omega }}^{\text{T}}\left(s\right)\tilde{\omega }\left(s\right)\right)\text{d}s\\ \leq \mu_{\sigma \left(t\right)}e^{\lambda_{\sigma \left(t\right)}\left(t-t_{k}\right)}\left(e^{\lambda_{\sigma \left(t_{k}\right)}\left(t_{k}-t_{k-1}\right)}V_{\sigma \left(t_{k}\right)}\left(t_{k-1}\right)\right.\\ \left.-\int_{t_{k-1}}^{t_{k}}e^{\lambda_{\sigma \left(t\right)}\left(t_{k}-s\right)}\left({\tilde{r}}^{\text{T}}\left(s\right)\tilde{r}\left(s\right)-\gamma^{2}{\tilde{\omega }}^{\text{T}}\left(s\right)\tilde{\omega }\left(s\right)\right)\text{d}s\right)\\ -\int_{t_{k}}^{t}e^{\lambda_{\sigma \left(t\right)}\left(t-s\right)}\left({\tilde{r}}^{\text{T}}\left(s\right)\tilde{r}\left(s\right)-\gamma^{2}{\tilde{\omega }}^{\text{T}}\left(s\right)\tilde{\omega }\left(s\right)\right)\text{d}s\\ \leq \mu_{\sigma \left(t\right)}\mu_{\sigma \left(t_{k}\right)}e^{\lambda_{\sigma \left(t\right)}\left(t-t_{k}\right)}e^{\lambda_{\sigma \left(t_{k}\right)}\left(t_{k}-t_{k-1}\right)}V_{\sigma \left(t_{k-1}\right)}\left(t_{k-1}^{-}\right)\\ -\mu_{\sigma \left(t\right)}e^{\lambda_{\sigma \left(t\right)}\left(t-t_{k}\right)}\int_{t_{k-1}}^{t_{k}}e^{\lambda_{\sigma \left(t\right)}\left(t_{k}-s\right)}\left({\tilde{r}}^{\text{T}}\left(s\right)\tilde{r}\left(s\right)-\gamma^{2}{\tilde{\omega }}^{\text{T}}\left(s\right)\tilde{\omega }\left(s\right)\right)\text{d}s\\ -\int_{t_{k}}^{t}e^{\lambda_{\sigma \left(t\right)}\left(t-s\right)}\left({\tilde{r}}^{\text{T}}\left(s\right)\tilde{r}\left(s\right)-\gamma^{2}{\tilde{\omega }}^{\text{T}}\left(s\right)\tilde{\omega }\left(s\right)\right)\text{d}s\\ \cdot \cdot \cdot \\ \leq \prod_{s=1}^{k}\mu_{\sigma \left(t_{s}\right)}e^{\sum_{s=0}^{k}\lambda_{\sigma \left(t_{s}\right)}\left(t_{s+1}-t_{s}\right)}V_{\sigma \left(0\right)}\left(0\right)\\ -\int_{t_{k}}^{t}e^{\lambda_{\sigma \left(t\right)}\left(t-s\right)}\left({\tilde{r}}^{\text{T}}\left(s\right)\tilde{r}\left(s\right)-\gamma^{2}{\tilde{\omega }}^{\text{T}}\left(s\right)\tilde{\omega }\left(s\right)\right)\text{d}s\\ -\mu_{\sigma \left(t\right)}e^{\lambda_{\sigma \left(t\right)}\left(t-t_{k}\right)}\int_{t_{k-1}}^{t_{k}}e^{\lambda_{\sigma \left(t\right)}\left(t_{k}-s\right)}\left({\tilde{r}}^{\text{T}}\left(s\right)\tilde{r}\left(s\right)-\gamma^{2}{\tilde{\omega }}^{\text{T}}\left(s\right)\tilde{\omega }\left(s\right)\right)\text{d}s\\ \cdot \cdot \cdot \\ -\prod_{s=1}^{k}\mu_{\sigma \left(t_{s}\right)}e^{\sum_{s=1}^{k}\lambda_{\sigma \left(t_{s}\right)}\left(t_{s+1}-t_{s}\right)}\int_{0}^{t_{1}}e^{\lambda_{\sigma \left(t\right)}\left(t_{1}-s\right)}\left({\tilde{r}}^{\text{T}}\left(s\right)\tilde{r}\left(s\right)-\gamma^{2}{\tilde{\omega }}^{\text{T}}\left(s\right)\tilde{\omega }\left(s\right)\right)\text{d}s\\ \leq \exp\left[\sum_{i=1}^{k}N_{\sigma ,i}\left(0,t\right)\ln\mu_{\sigma \left(t_{i}\right)}+\sum_{i=1}^{k}T_{i}\left(0,t\right)\lambda_{\sigma \left(t_{i}\right)}\right]V_{\sigma \left(0\right)}\left(0\right)\\ -\int_{0}^{t}\exp\left[\sum_{i=1}^{k}N_{\sigma ,i}\left(s,t\right)\ln\mu_{\sigma \left(t_{i}\right)}+\sum_{i=1}^{k}T_{i}\left(s,t\right)\lambda_{\sigma \left(t_{i}\right)}\right]\left({\tilde{r}}^{\text{T}}\left(s\right)\tilde{r}\left(s\right)-\gamma^{2}{\tilde{\omega }}^{\text{T}}\left(s\right)\tilde{\omega }\left(s\right)\right)\text{d}s. \end{array}$$

Together with the condition *V*_*σ*(*t*)_(*t*) ≥ 0, one can conclude

(26) $$\begin{array}{c} \int_{0}^{t}\exp\left[\sum_{i=1}^{k}N_{\sigma ,i}\left(s,t\right)\ln\mu_{\sigma \left(t_{i}\right)}+\sum_{i=1}^{k}T_{i}\left(s,t\right)\lambda_{\sigma \left(t_{i}\right)}\right]\left({\tilde{r}}^{\text{T}}\left(s\right)\tilde{r}\left(s\right)-\gamma^{2}{\tilde{\omega }}^{\text{T}}\left(s\right)\tilde{\omega }\left(s\right)\right)\text{d}s\leq 0\\ \Leftrightarrow \int_{0}^{t}\exp\left[\sum_{i=1}^{k}N_{\sigma ,i}\left(s,t\right)\ln\mu_{\sigma \left(t_{i}\right)}+\sum_{i=1}^{k}T_{i}\left(s,t\right)\lambda_{\sigma \left(t_{i}\right)}\right]{\tilde{r}}^{\text{T}}\left(s\right)\tilde{r}\left(s\right)\text{d}s\\ \leq \gamma^{2}\int_{0}^{t}\exp\left[\sum_{i=1}^{k}N_{\sigma ,i}\left(s,t\right)\ln\mu_{\sigma \left(t_{i}\right)}+\sum_{i=1}^{k}T_{i}\left(s,t\right)\lambda_{\sigma \left(t_{i}\right)}\right]{\tilde{\omega }}^{\text{T}}\left(s\right)\tilde{\omega }\left(s\right)\text{d}s. \end{array}$$

Multiplying both sides of equation (26) by exp[−∑_*i*=1_^*k*^*N*_*σ*,*i*_(0, *t*)ln*μ*_*σ*(*t*_*i*_)_], we can obtain

(27) $$\begin{array}{c} \int_{0}^{t}\exp\left[-\sum_{i=1}^{k}N_{\sigma ,i}\left(0,s\right)\ln\mu_{\sigma \left(t_{i}\right)}+\sum_{i=1}^{k}T_{i}\left(s,t\right)\lambda_{\sigma \left(t_{i}\right)}\right]{\tilde{r}}^{\text{T}}\left(s\right)\tilde{r}\left(s\right)\text{d}s\\ \leq \gamma^{2}\int_{0}^{t}\exp\left[-\sum_{i=1}^{k}N_{\sigma ,i}\left(0,s\right)\ln\mu_{\sigma \left(t_{i}\right)}+\sum_{i=1}^{k}T_{i}\left(s,t\right)\lambda_{\sigma \left(t_{i}\right)}\right]{\tilde{\omega }}^{\text{T}}\left(s\right)\tilde{\omega }\left(s\right)\text{d}s. \end{array}$$

Together with equation (21) and the condition in equation (28), we can obtain equation (29).

(28) $$\begin{array}{c} N_{\sigma ,i}\left(0,s\right)\ln\mu_{\sigma \left(t_{i}\right)}\leq \frac{T_{i}\left(0,s\right)}{\tau_{ai}}\ln\mu_{\sigma \left(t_{i}\right)}\leq -T_{i}\left(0,s\right)\lambda_{\sigma \left(t_{i}\right)}, \end{array}$$

(29) $$\begin{array}{c} \int_{0}^{t}\exp\left[\sum_{i=1}^{k}T_{i}\left(0,s\right)\lambda_{\sigma \left(t_{i}\right)}+\sum_{i=1}^{k}T_{i}\left(s,t\right)\lambda_{\sigma \left(t_{i}\right)}\right]{\tilde{r}}^{\text{T}}\left(s\right)\tilde{r}\left(s\right)\text{d}s\\ \leq \int_{0}^{t}\exp\left[-\sum_{i=1}^{k}N_{\sigma ,i}\left(0,s\right)\ln\mu_{\sigma \left(t_{i}\right)}+\sum_{i=1}^{k}T_{i}\left(s,t\right)\lambda_{\sigma \left(t_{i}\right)}\right]{\tilde{r}}^{\text{T}}\left(s\right)\tilde{r}\left(s\right)\text{d}s\\ \leq \gamma^{2}\int_{0}^{t}\exp\left[-\sum_{i=1}^{k}N_{\sigma ,i}\left(0,s\right)\ln\mu_{\sigma \left(t_{i}\right)}+\sum_{i=1}^{k}T_{i}\left(s,t\right)\lambda_{\sigma \left(t_{i}\right)}\right]{\tilde{\omega }}^{\text{T}}\left(s\right)\tilde{\omega }\left(s\right)\text{d}s\\ \leq \gamma^{2}\int_{0}^{t}\exp\left[-\sum_{i=1}^{k}N_{\sigma ,i}\left(0,t\right)\ln\mu_{\sigma \left(t_{i}\right)}+\sum_{i=1}^{k}T_{i}\left(s,t\right)\lambda_{\sigma \left(t_{i}\right)}\right]{\tilde{\omega }}^{\text{T}}\left(s\right)\tilde{\omega }\left(s\right)\text{d}s\\ \leq \gamma^{2}e^{-\sum_{i=1}^{k}N_{\sigma ,i}\left(0,t\right)\ln\mu_{\min}}\int_{0}^{t}\exp\left[\sum_{i=1}^{k}T_{i}\left(s,t\right)\lambda_{\sigma \left(t_{i}\right)}\right]{\tilde{\omega }}^{\text{T}}\left(s\right)\tilde{\omega }\left(s\right)\text{d}s\\ \leq \gamma^{2}\mu_{\min}^{-k}\int_{0}^{t}\exp\left[\sum_{i=1}^{k}T_{i}\left(s,t\right)\lambda_{\sigma \left(t_{i}\right)}\right]{\tilde{\omega }}^{\text{T}}\left(s\right)\tilde{\omega }\left(s\right)\text{d}s\\ \leq \gamma^{2}\mu_{\min}^{-k}\int_{0}^{t}\exp\left[\sum_{i=1}^{k}T_{i}\left(0,t\right)\lambda_{\sigma \left(t_{i}\right)}\right]{\tilde{\omega }}^{\text{T}}\left(s\right)\tilde{\omega }\left(s\right)\text{d}s, \end{array}$$

and it can be inferred that

(30) $$\begin{array}{c} \int_{0}^{t}\exp\left[\sum_{i=1}^{k}T_{i}\left(0,t\right)\lambda_{\sigma \left(t_{i}\right)}\right]{\tilde{r}}^{\text{T}}\left(s\right)\tilde{r}\left(s\right)\text{d}s\leq \gamma^{2}\mu_{\min}^{-k}\int_{0}^{t}\exp\left[\sum_{i=1}^{k}T_{i}\left(0,t\right)\lambda_{\sigma \left(t_{i}\right)}\right]{\tilde{\omega }}^{\text{T}}\left(s\right)\tilde{\omega }\left(s\right)\text{d}s\\ \int_{0}^{t}{\tilde{r}}^{\text{T}}\left(s\right)\tilde{r}\left(s\right)\text{d}s\leq \gamma^{2}\mu_{\min}^{-k}\int_{0}^{t}{\tilde{\omega }}^{\text{T}}\left(s\right)\tilde{\omega }\left(s\right)\text{d}s. \end{array}$$

Integrating both sides of equation (30) from 0 to *∞*, we have

(31) $$\begin{array}{c} \int_{0}^{\infty }{\tilde{r}}^{\text{T}}\left(s\right)\tilde{r}\left(s\right)\text{d}s\leq \gamma^{2}\mu_{\min}^{-k}\int_{0}^{\infty }{\tilde{\omega }}^{\text{T}}\left(s\right)\tilde{\omega }\left(s\right)\text{d}s. \end{array}$$

We can conclude that the switched system in equation (5) is GUES with prescribed attenuation performance *γ*_*d*_=*γ*^2^*μ*_min_^−*k*/2^.

**Theorem 3 .**

For given constant scalars 0 < *μ*_*i*_ < 1, *λ*_*i*_ > 0, and *γ* > 0, if there exist positive definite matrices *P*_*i*_, such that

(32) $$\begin{array}{c} P_{i}\leq \mu_{i}P_{j},\forall i\neq j,\\ \left[\begin{array}{ccccc} \Phi_{i} & P_{i}{\tilde{B}}_{i} & {\tilde{C}}_{i}^{\text{T}} & \Psi_{1i} & \Psi_{2i}\\ {}* & -\gamma^{2}I & {\tilde{D}}_{i}^{\text{T}} & 0 & 0\\ {}* & * & -I & 0 & 0\\ {}* & * & * & W_{i} & 0\\ {}* & * & * & * & W_{i} \end{array}\right]\leq 0. \end{array}$$

Then, the switched system with MDADT satisfying equation (16) is GUES with prescribed attenuation performance *γ*_*d*_; the parameters of fault detection filter can be given by

(33) $$\begin{array}{c} L_{ni}=P_{2i}^{-1}X_{1i}, \end{array}$$

where $P_{i}=\left[\begin{array}{cc} P_{1i} & P_{2i}\\ {}* & P_{3i} \end{array}\right]$, $\Phi_{i}=\left[\begin{array}{cc} A_{i}{}^{\text{T}}P_{1i}+P_{1i}A_{i}-\lambda P_{1i}, & A_{i}{}^{\text{T}}P_{2i}+P_{2i}A_{i}-\lambda P_{2i}-X_{1i}C_{i},\\ {}*, & A_{i}{}^{\text{T}}P_{3i}+P_{3i}A_{i}-\lambda P_{3i}-X_{2i}C_{i}-C_{i}{}^{\text{T}}X_{2i}^{\text{T}}, \end{array}\right]$, *X*_1*i*_=*P*_2*i*_*L*_*ni*_, *X*_2*i*_=*P*_3*i*_*L*_*ni*_, $\Psi_{1i}=\left[\begin{array}{cc} \Theta_{1i}^{\text{T}} & \Theta_{2i}^{\text{T}} \end{array}\right]$, $\Psi_{2i}=\left[\begin{array}{cc} \Theta_{3i}^{\text{T}} & \Theta_{4i}^{\text{T}} \end{array}\right]$, $\Theta_{1i}=\left[\begin{array}{cc} -M_{1i}^{\text{T}}P_{2i}^{\text{T}} & 0 \end{array}\right]$, $\Theta_{2i}=\left[\begin{array}{cc} 0 & N_{1i}C_{i} \end{array}\right]$, $\Theta_{3i}=\left[\begin{array}{cc} 0 & -M_{2i}^{\text{T}}P_{3i}^{\text{T}} \end{array}\right]$, $\Theta_{4i}=\left[\begin{array}{cc} 0 & N_{2i}C_{i} \end{array}\right]$, $W_{i}=\left[\begin{array}{cc} -\kappa_{i} & 0\\ 0 & -\kappa_{i}^{-1} \end{array}\right]$.

**Proof**

It can be inferred that equation (14) can be rewritten as

(34) $$\begin{array}{c} Y_{i}+S_{1i}{}^{\text{T}}E_{i}T_{1i}+T_{1i}{}^{\text{T}}E_{i}{}^{\text{T}}S_{1i}+S_{2i}{}^{\text{T}}E_{i}T_{2i}+T_{2i}{}^{\text{T}}E_{i}{}^{\text{T}}S_{2i}\leq 0, \end{array}$$

where $Y_{i}=\left[\begin{array}{ccc} {\tilde{A}}_{1i}^{\text{T}}P_{i}+P_{i}{\tilde{A}}_{1i}-\lambda_{i}P_{i} & P_{i}{\tilde{B}}_{i} & {\tilde{C}}_{i}^{\text{T}}\\ {}* & -\gamma^{2}I & {\tilde{D}}_{i}^{\text{T}}\\ {}* & * & -I \end{array}\right]$, ${\tilde{A}}_{1i}=\left[\begin{array}{cc} A_{i} & 0\\ 0 & A_{i}-L_{ni}C_{i} \end{array}\right]$, $S_{1i}=\left[\begin{array}{ccc} \Theta_{1i} & 0 & 0 \end{array}\right]$, $T_{1i}=\left[\begin{array}{ccc} \Theta_{2i} & 0 & 0 \end{array}\right]$$S_{2i}=\left[\begin{array}{ccc} \Theta_{3i} & 0 & 0 \end{array}\right]$, $T_{2i}=\left[\begin{array}{ccc} \Theta_{4i} & 0 & 0 \end{array}\right]$.

Based on Lemma 1, we have

(35) $$\begin{array}{c} Y_{i}+\kappa^{-1}S_{1i}{}^{\text{T}}S_{1i}+\kappa T_{1i}{}^{\text{T}}T_{1i}+\kappa^{-1}S_{2i}{}^{\text{T}}S_{2i}+\kappa T_{2i}{}^{\text{T}}T_{2i}<0. \end{array}$$

Thus, by the aid of Schur complement, we can conclude that the switched system in equation (5) is GUES with prescribed performance *γ*_*d*_; the parameters of fault detection filters can be obtained by equation (33).

**Remark 3 .**

The learning-based fault detection filter is viewed as the compensation of the robust filter. Therefore, we can obtain the scheduling interval to ensure the stability and prescribed attenuation performance by the aid of nonfragile control theory in Theorem 3. The upper bounds on action in DDPG algorithm can be obtained by the predefined scheduling interval.

### 3.2. Intelligent Filter Design

The stability and prescribed attenuation performance are guaranteed by the theorems aforementioned. However, the transient performance needs to be improved. To solve the problem, deep reinforcement learning is applied in the framework of actor-critic. The optimization of parameters of fault detection filter can be viewed as an infinite Markov decision process, which is a series of continuous optimization processes. Thus, the DDPG algorithm is developed in this paper to improve the performance of the filter.

The frame of reinforcement learning consists of an agent and the environment. The state at *k*th time instant is defined as *s*_*k*_, an action *a*_*k*_ is chosen by the agent, and then a reward function *r*_*k*_ and the state of next step are obtained, where *r*_*k*_ is developed to evaluate the performance of state-action pair generated by the agent. The fault detection system is viewed as the environment. We define the additional compensation *L*_d,*σ*(*t*)_ as the action, which is utilized to maximize the sum of the expected discounted reward function over given future steps. The action and the sum of expected discounted reward function can be described in equations (36) and (37).

(36) $$\begin{array}{c} R\left(k\right)=r_{e,k}+\gamma_{f}r_{e,k+1}+\gamma_{f}^{2}r_{e,k+2}+\cdots +\gamma_{f}^{K_{f}-k}r_{e,K_{f}}=r_{e,k}+\gamma_{f}R\left(k+1\right), \end{array}$$

(37) $$\begin{array}{c} a_{k}=\Delta L_{i}, \end{array}$$

where *γ*_*f*_ ∈ [0,1] is defined as the discount factor and *K*_*f*_ represents the terminal step.

The state of the agent is given in the following equation:

(38) $$\begin{array}{c} s_{k}=\left[\begin{array}{cccc} x\left(k\right) & e\left(k\right) & r\left(k\right) & \tilde{r}\left(k\right) \end{array}\right]. \end{array}$$

The DDPG algorithm is proposed in the framework of deep *Q* learning and actor-critic. There are two actor networks and two critic networks. The optimal policy is tried for realization based on policy gradient theory in continuous action spaces. The action-value is approximated by employing the critic network *Q*(*s*_*k*_, *a*_*k*_*|ϖ*^*Q*^), whose weights are defined as *ϖ*^*Q*^. The current output of compensated parameters is generated based on the actor network *a*(*s*_*k*_*|ϖ*^*a*^), whose weights are defined as *ϖ*^*a*^. The weights *ϖ*^*Q*^ are updated according to the loss function, which is described in equation the following:

(39) $$\begin{array}{c} L\left(\varpi^{Q}\right)=E_{\left(s,a\right)}\left[{\left(Q\left(s_{k},a_{k}|\varpi^{Q}\right)-{\bar{y}}_{k}\right)}^{2}\right], \end{array}$$

where ${\bar{y}}_{k}=r_{k}\left(s_{k},a_{k}\right)+\gamma_{p}Q\left(s_{k+1},\varpi \left(s_{k}|\varpi^{a}\right)|\varpi^{Q}\right)$.

The weights of actor network are updated according to the policy gradient theory, which is given in equations (40) and (41).

(40) $$\begin{array}{c} \varpi^{a}\left(k+1\right)=\varpi^{a}\left(k\right)+L_{an}\nabla_{\varpi^{a}}J, \end{array}$$

(41) $$\begin{array}{c} \nabla_{\varpi^{a}}J={\text{E}}_{\pi }\left[{\nabla_{\varpi^{a}}Q^{\pi }\left(s_{k},\pi \left(s_{k}|\varpi^{a}\right)|\varpi^{Q}\right)|}_{s=s_{k},a=\pi \left(s_{k}|\varpi^{a}\right)}\right] \end{array}$$

where *L*_*an*_ represents the learning rate of actor network.

Moreover, in the DDPG algorithm, two networks are adopted as actor target network and critic target network, which are defined as *a*′(*s*_*k*_*|ϖ*^*a*′^) and *Q*′(*s*_*k*_, *a*_*k*_*|ϖ*^*Q*′^). The weights of actor target network *a*′(*s*_*k*_*|ϖ*^*a*′^) are defined as *ϖ*^*a*′^, which are updated according to the following equation:

(42) $$\begin{array}{c} \varpi^{a\prime }\left(k+1\right)=L_{at}\varpi^{a}\left(k\right)+\left(1-L_{at}\right)\varpi^{a^{\prime }}\left(k\right). \end{array}$$

*L*_*at*_ denotes the learning rate of actor target network.

Similarly, the weights of critic target network *Q*′(*s*_*k*_, *a*_*k*_*|ϖ*^*Q*′^) are defined as *ϖ*^*Q*′^, which are updated according to the following equation:

(43) $$\begin{array}{c} \varpi^{Q^{\prime }}\left(k+1\right)=L_{ct}\varpi^{Q}\left(k+1\right)+\left(1-L_{ct}\right)\varpi^{Q^{\prime }}\left(k+1\right). \end{array}$$

*L*_*ct*_ represents the learning rate of critic target network.

In order to improve the robustness of the proposed algorithm, an exploration noise is introduced as a compensation of the output of actor network, which can be generated based on

(44) $$\begin{array}{c} a_{k}=\pi \left(s_{k}|\varpi^{a}\right)+N_{a}. \end{array}$$

Therefore, based on the statement above, the pseudocode of intelligent fault detection filters design can be presented in Algorithm 1.

**Remark 4 .**

The DDPG algorithm is developed in this paper to improve the transient performance of the fault detection filter. Compared with the traditional method, the stability, robustness, and dynamic performance can be guaranteed simultaneously, in which the robust control theory and nonfragile control theory are introduced to ensure the stability, and the compensation of controller can be viewed as the variation of predesigned controller. Therefore, the stability of closed-loop system can be guaranteed by nonfragile control theory.

## 4. Numerical Example

In this section, simulation results are given to validate the effectiveness of the proposed method. The system matrices are given as follows:

(45) $$\begin{array}{c} {\text{A}}_{1}=\left[\begin{array}{cc} -2.23 & -3.72\\ 2.83 & 2.32 \end{array}\right],\\ {\text{A}}_{2}=\left[\begin{array}{cc} -1.25 & 1.26\\ 2.22 & -2.22 \end{array}\right],\\ {\text{C}}_{1}=\left[\begin{array}{cc} 1 & 0\\ 0 & 1 \end{array}\right],\\ {\text{C}}_{2}=\left[\begin{array}{cc} 1 & 0\\ 0 & 1 \end{array}\right],\\ {\text{D}}_{1}=\left[\begin{array}{c} 0.1\\ 0.1 \end{array}\right],\\ {\text{D}}_{2}=\left[\begin{array}{c} 0.2\\ 0.2 \end{array}\right],\\ {\text{F}}_{1}=\left[\begin{array}{c} 0.1\\ 0.2 \end{array}\right],\\ {\text{F}}_{2}=\left[\begin{array}{c} 0.1\\ 0.1 \end{array}\right]. \end{array}$$

The eigenvalues of ***A***_1_ and ***A***_2_ can be obtained as follows:

(46) $$\begin{array}{c} \left\{\begin{array}{c} \lambda_{11}=0.045+2.3134i,\\ \lambda_{12}=0.045-2.3134i, \end{array}\right.\left\{\begin{array}{c} \lambda_{21}=0.0064,\\ \lambda_{22}=-3.4764. \end{array}\right. \end{array}$$

It can be inferred that the subsystems ***A***_1_ and ***A***_2_ are unstable, respectively. The external disturbance is defined as follows:

(47) $$\begin{array}{c} \omega \left(t\right)=0.1\exp\left(-0.1t\right)\cos\left(-0.1t\right). \end{array}$$

The other parameters of switched systems are listed as follows:

(48) $$\begin{array}{c} \left\{\begin{array}{c} \mu_{1}=0.73,\\ \mu_{2}=0.73, \end{array}\right.\left\{\begin{array}{c} \lambda_{1}=0.62,\\ \lambda_{2}=0.65. \end{array}\right. \end{array}$$

Therefore, the MDADT of switched systems are *τ*_1_=0.5076, *τ*_2_=0.4842. The prescribed attenuation performance index is set to be *γ*=0.8. Then the parameter matrices of filters can be obtained in Theorem 3.

To validate the effectiveness of the proposed method, we give two numerical examples. The stability criteria of the switched systems in Theorem 2 are demonstrated by Example 1. Moreover, the effectiveness of the fault detection filter in Theorem 3 and the DDPG algorithm is illustrated in Example 2.

**Example 1 .**

**Figure 1:**
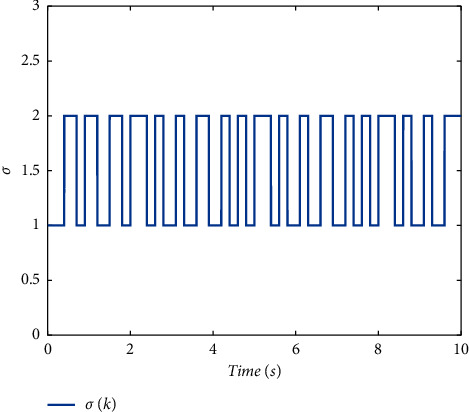
The switching logic satisfying equation (20).

**Figure 2:**
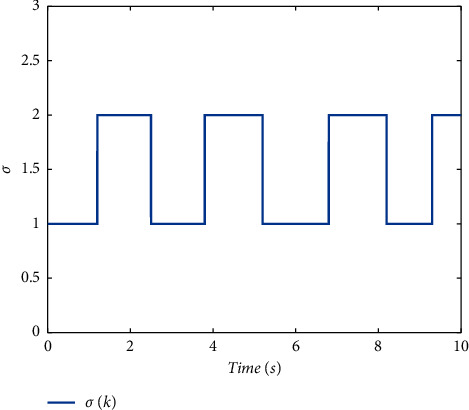
A randomly generated switching signal.

**Figure 3:**
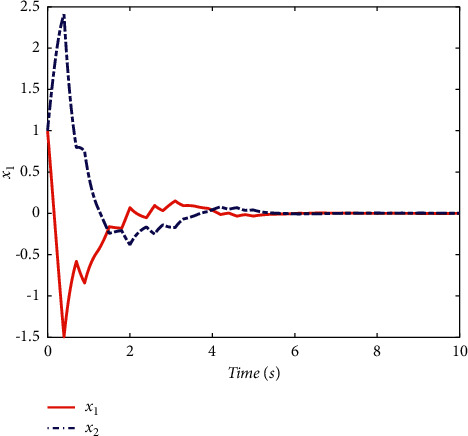
The state response of the proposed switching logic.

**Figure 4:**
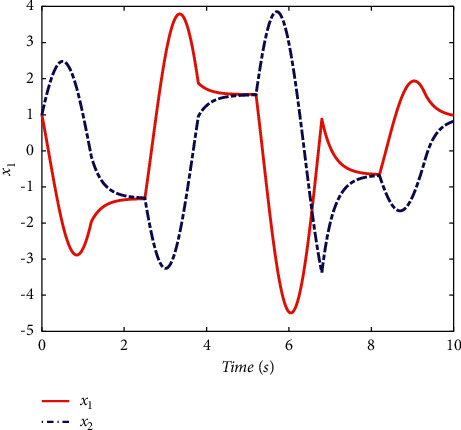
The state response of randomly generated switching logic.

Firstly, we provide the proof that the proposed conditions can ensure the stability of switched systems. As a comparison, a randomly generated switching signal is introduced to show that the switched systems cannot stay stable if all the subsystems cannot share a CLF. The switching logic satisfying equation (20) is given in Figure 1 and the randomly generated switching logic is given in Figure 2. The state response of the proposed switching logic is given in Figure 3 and the state response of randomly generated switching logic is showed in Figure 4. We can see that the switched systems cannot ensure the stability under randomly generated switching logic. However, the stability can be guaranteed according to Theorem 1 in the paper.

**Example 2 .**

To validate the effectiveness of the fault detection filter, the traditional MDADT method and the proposed method are given. The fault signal is set to be

(49) $$\begin{array}{c} \text{f}\left(k\right)=\left\{\begin{array}{cc} 0.5, & 4\leq t\leq 5,\\ 0, & \text{else.} \end{array}\right. \end{array}$$

**Figure 5:**
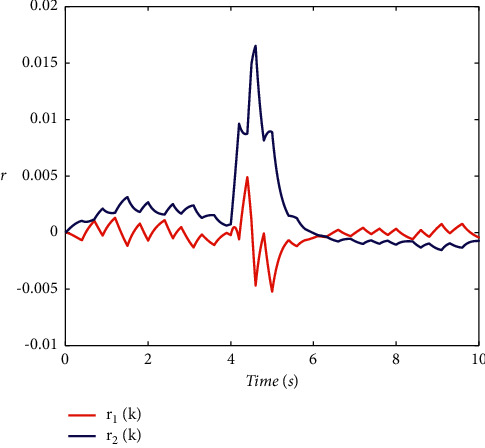
The response of residual signal under MDADT method.

**Figure 6:**
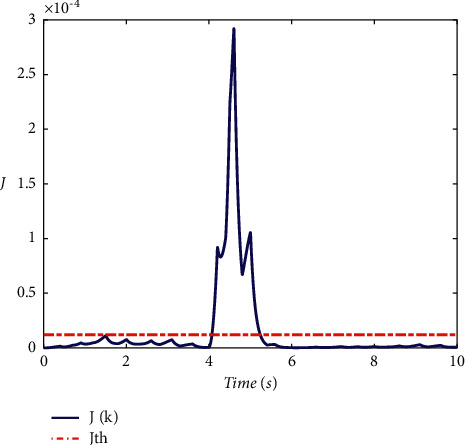
The response of *J*(*k*) under MDADT method.

**Figure 7:**
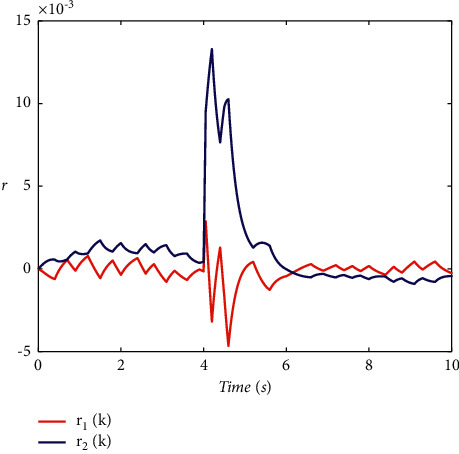
The response of residual signal under the proposed method.

**Figure 8:**
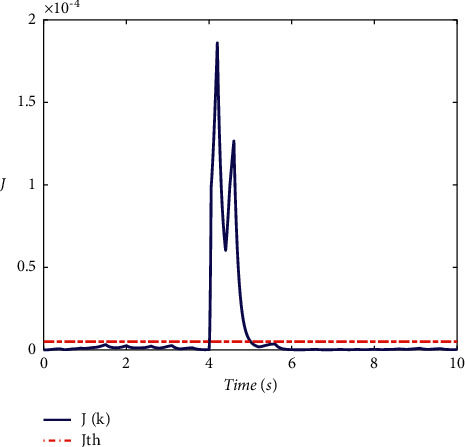
The response of *J*(*k*) under the proposed method.

**Figure 9:**
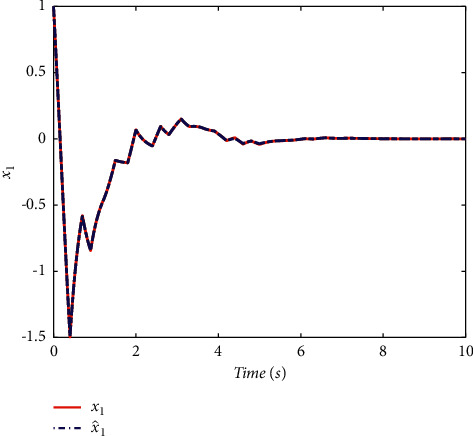
The state response of the MDADT method.

**Figure 10:**
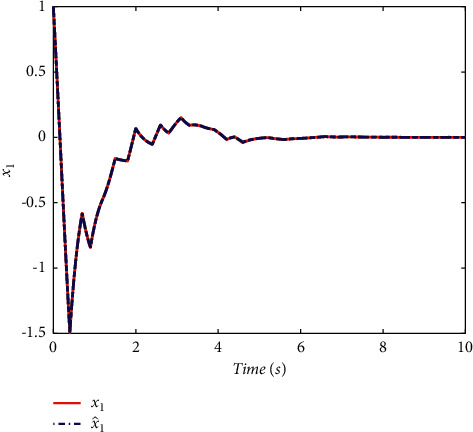
The state response of the proposed method.

**Figure 11:**
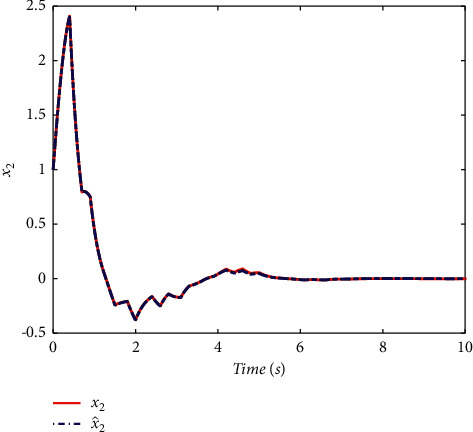
The state response of MDADT method under fault case.

**Figure 12:**
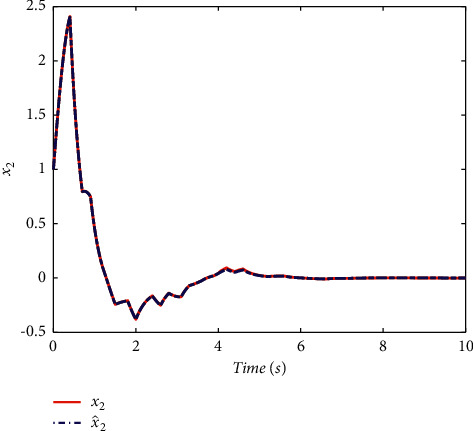
The state response of the proposed method under fault case.

**Figure 13:**
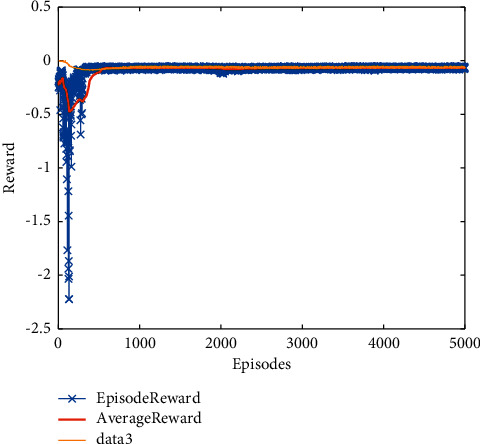
The response of episode reward.

The results are given in Figures 5–12. The residual signal and *J*(*k*) of MDADT method are given in Figures 5 and 6; the residual signal and *J*(*k*) of the proposed method are showed in Figures 7 and 8. We can see that the detection time of the MDADT method is 0.1 s, whereas, the detection time of the proposed method is 0.06 s. The fault can be detected efficiently, and the transient performance of fault detection filter can be improved by the DDPG algorithm. The state responses of the MDADT method and the proposed method are depicted in Figures 9–12. It can be inferred that the fault detection filter proposed in this paper can track the state response. The response of episodes reward is showed in Figure 13; we can see that the reward function can converge to a neighbor of the origin, which illustrate the effectiveness of the optimization algorithm.

In summary, we can see that the tighter bounds on dwell time and less conservative results are obtained. The stability of switched systems can be guaranteed by the proposed results in this paper. The fast switching strategy is applied, and it allows the subsystems of switched systems to remain unstable. The transient performance of fault detection filters can be improved by the aid of DDPG algorithm, and the stability, robustness, and optimal policy can be guaranteed simultaneously by the method proposed in this paper.

## 5. Conclusions

The problem of fault detection and online scheduling for switched systems with all modes unstable is studied. The observer-based fault detection filter is proposed to generate the residual signal, which consists of two parts: the dynamics-based fault detection filter and the learning-based fault detection filter. By employing MDADT method and MLF method, the stability of the switched systems is guaranteed. The solutions of fault detection filters are given in the form of LMIs. To achieve optimal control policy and improve the transient performance, the DDPG algorithm is utilized as learning-based fault detection filter, in which the output can be viewed as the variation of robust fault detection filter. Therefore, the nonfragile control is provided to ensure the stability of the optimization algorithm. Finally, the simulation results are introduced to demonstrate the effectiveness and superiority of the proposed method.

## Figures and Tables

**Algorithm 1:**
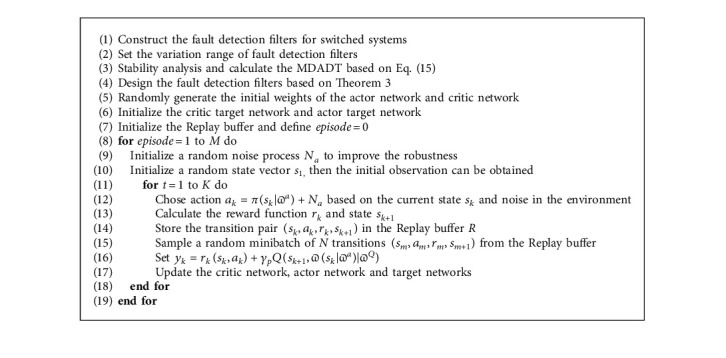
Intelligent optimization algorithm for fault detection filters design.

## Data Availability

The data used to support the findings of this study are included within the article.
